# *N*-[(2-Hydr­oxy-5-methoxy­phen­yl)(3-nitro­phen­yl)meth­yl]acetamide

**DOI:** 10.1107/S1600536809009726

**Published:** 2009-03-25

**Authors:** M. NizamMohideen, S. Thenmozhi, A. SubbiahPandi, N. Panneer Selvam, P. T. Perumal

**Affiliations:** a Department of Physics, The New College (Autonomous), Chennai 600 014, India; b Department of Physics, Presidency College (Autonomous), Chennai 600 005, India; c Organic Chemistry Division, Central Leather Research Institute, Chennai 600 020, India

## Abstract

In the title compound, C_16_H_16_N_2_O_5_, the meth­oxy group is disordered with site occupancies of 0.20 (3) and 0.80 (3). The dihedral angle between the two aromatic rings is 73.7 (2)°. The crystal structure is characterized by intermolecular N—H⋯O, O—H⋯O, C—H⋯O and C—H⋯π hydrogen bonds.

## Related literature

For *N*-substituted phen­yl acetamides as inter­mediates in organic synthesis, see: Gowda *et al.* (2007 ▶); Ghosh *et al.* (2005 ▶). For a related structure, see: NizamMohideen *et al.* (2009 ▶). For hydrogen-bond motifs, see: Bernstein *et al.* (1995 ▶).
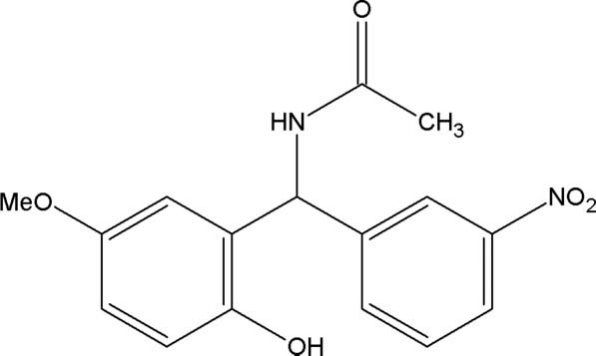


## Experimental

### 

#### Crystal data

C_16_H_16_N_2_O_5_
*M* *_r_* = 316.31
Monoclinic, 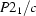

*a* = 15.3351 (3) Å
*b* = 8.1327 (2) Å
*c* = 14.5308 (3) Å
β = 117.387 (1)°
*V* = 1609.10 (6) Å^3^
*Z* = 4
Mo *K*α radiation
μ = 0.10 mm^−1^
*T* = 293 K
0.32 × 0.28 × 0.25 mm

#### Data collection

Bruker Kappa APEXII CCD diffractometer
Absorption correction: none
23127 measured reflections
6121 independent reflections
3900 reflections with *I* > 2σ(*I*)
*R*_int_ = 0.032

#### Refinement

*R*[*F* ^2^ > 2σ(*F* ^2^)] = 0.058
*wR*(*F* ^2^) = 0.196
*S* = 1.03
6121 reflections
216 parameters
1 restraint
H-atom parameters constrained
Δρ_max_ = 0.41 e Å^−3^
Δρ_min_ = −0.25 e Å^−3^

### 

Data collection: *APEX2* (Bruker, 2004 ▶); cell refinement: *APEX2* and *SAINT* (Bruker, 2004 ▶); data reduction: *SAINT* and *XPREP* (Bruker, 2004 ▶); program(s) used to solve structure: *SHELXS97* (Sheldrick, 2008 ▶); program(s) used to refine structure: *SHELXL97* (Sheldrick, 2008 ▶); molecular graphics: *ORTEP-3* (Farrugia, 1997 ▶); software used to prepare material for publication: *SHELXL97* and *PLATON* (Spek, 2009 ▶).

## Supplementary Material

Crystal structure: contains datablocks global, I. DOI: 10.1107/S1600536809009726/bt2901sup1.cif

Structure factors: contains datablocks I. DOI: 10.1107/S1600536809009726/bt2901Isup2.hkl

Additional supplementary materials:  crystallographic information; 3D view; checkCIF report

## Figures and Tables

**Table 1:** Hydrogen-bond geometry (Å, °)

*D*—H⋯*A*	*D*—H	H⋯*A*	*D*⋯*A*	*D*—H⋯*A*
O2—H2⋯O5^i^	0.82	1.80	2.617 (2)	179
N1—H1⋯O4^ii^	0.86	2.30	3.159 (2)	174
C10—H10⋯O1^iii^	0.93	2.47	3.320 (2)	152
C12—H12⋯O2^iv^	0.93	2.58	3.397 (2)	147
C14—H14⋯O4^ii^	0.93	2.55	3.470 (2)	169
C8—H8⋯O5	0.98	2.30	2.714 (2)	105
C11—H11⋯*Cg*1^iv^	0.93	2.83	3.680 (2)	153
C1*B*—H1*B*1⋯*Cg*2^v^	0.96	2.61	3.531 (2)	160

Symmetry codes: (i) 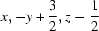; (ii) 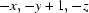; (iii) 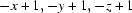; (iv) 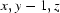; (v) 
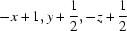
. *Cg*1 and *Cg*2 are the centroids of the C2-C7 and C9-C14 rings, respectively.

**Fig. 1.**
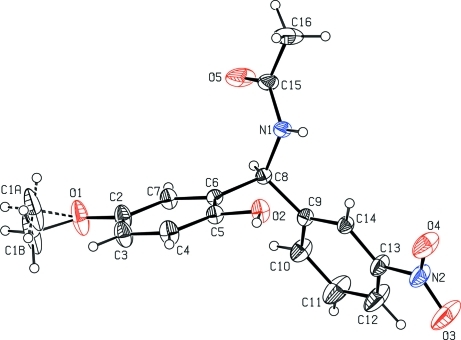
View of the title compound showing the atom-numbering scheme. Displacement ellipsoids are drawn at the 30% probability level.

**Fig. 2.**
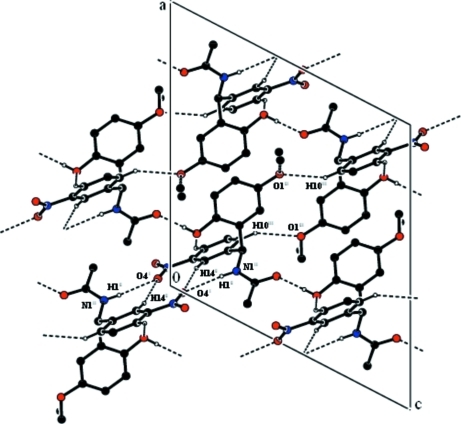
The crystal packing of the title compound showing the R22(16), R22(10) and R22(16) rings. Hydrogen bonding is shown as dashed lines. H atoms not involved in the hydrogen bonding have been omitted for clarity. [Symmetry codes: (ii) -x + 1, -y + 1, -z + 1, (iii)-x, -y + 1, -z]]

## supplementary crystallographic information

### Comment

N-(Substituted phenyl) acetamides are well known for their importance as intermediates in organic synthesis (Gowda *et al.*, 2007). Depending on the types of substitution at the α, β and keto-C atoms, and the conformational flexibility of the substituent groups, a variety of β-acetamido ketones offering the possibility of intermolecular interactions can be obtained (Ghosh *et al.*, 2005). The amide linkage [–NHC(O)-] is known to be strong enough to form and maintain protein architectures and has been utilized to create various molecular devices for a spectrum of purposes in organic chemistry. We have synthesized an amide system with an aromatic ring as a terminal group to determine how the rigid ring affects the conformational behavior. As part of our ongoing investigation of acetamide derivatives, the title compound  has been prepared and its crystal structure is presented here.

The bond lenghts and angles are comparable with *N*-[(3-Nitro-phenyl)-(2-hydroxy-napthalen-1-yl)-methyl]-acetamide (NizamMohideen *et al.*, 2009), a structure closely related to the title compound. The nitro group is slighty twisted out of the plane of the benzene ring, as indicated by O4—N2—C13—C14 and O3—N2—C13—C14 torsion angles of -8.6 (3) and 171.0 (2)°, respectively, and comparable with those in the previously reported structure mentioned above.

The dihedral angle between the C2—C7 and C9—C14 benzene rings is 73.7 (2)°. The dihedral angle between the acetamide residue and the benzene rings (C2—C7 and C9—C14) are 70.0 (1) and 37.4 (2)°, respectively.

The intermolecular aggregation of the molecules is determined by combination of N—H···O, C—H···O, O—H···O and C—H···π hydrogen bonds (Table 1). The crystal structure is characterized by intermolecular bifurcated acceptor hydrogen bonds between the benzene and acetamide groups (Fig. 2). Atom N1 and C14 in the molecule at (*x*, *y*, *z*) act as a hydrogen-bond donor *via* atom H1 and H14 to atom O4 in the molecule at (-*x*, 1 - *y*, -*z*). This intermolecular hydrogen bond links the molecule into dimers with a cyclic *R*^2^_2_(16) and *R*^2^_2_(10) (Bernstein *et al.*, 1995) ring system, respectively. Atom C10 in the molecule at (*x*, *y*, *z*) acts as a hydrogen-bond donor *via* atom H10 to atom O1 in the molecule at (1 - *x*, 1 - *y*, 1 - *z*). This intermolecular hydrogen bond links the molecule into dimers with a cyclic *R*^2^_2_(16) ring system. The crystal structure is further stabilized by C—H···π interactions involing rings C11—H11···*Cg*1 (*Cg*1 is the centroid of the C2—C7 ring) and C1—H1a···*Cg*2 (where *Cg*2 is the centroid of the C9—C14 ring).

### Experimental

A mixture of 3-nitrobenzaldehyde (10 mmol), 4-methoxyphenol (10 mmol) and iodine (0.4 mmol, 4 mol%) were mixed in acetonitrile (5 ml). To that suspension acetyl chloride (2.8 mmol, 0.2 ml) was added and the reaction mixture was stirred at room temperature for 5 h. After the completion of the reaction (as monitored by TLC), saturated sodium thiosulfate solution (5 ml) was added. The precipitated solid was filtered and dried. The dried sample was washed with diethyl ether (2 × 10 ml) and again dried. Single crystals of the title compound suitable for X-ray diffraction were obtained by slow evaporation of a solution in Ethanol.

### Refinement

The C atoms of the methoxy group are disordered over two positions with refined occupancies of 0.20 (3) and 0.80 (3). The corresponding bond distances involving the disordered atoms were restrained to be equal. H atoms were positioned geometrically, with N—H = 0.86, O—H = 0.82 and C—H = 0.93, 0.98 and 0.96 Å aromatic, methylene and methyl H, respectively, and were treated as riding on their parent atoms, with *U*_iso_(H) = *xU*_eq_(C, N), where *x* = 1.2 for all H atoms.

### Crystal data

**Table:**

C_16_H_16_N_2_O_5_	*F*(000) = 664
*M**_r_* = 316.31	*D*_x_ = 1.306 Mg m^−^^3^
Monoclinic, *P*2_1_/*c*	Mo *K*α radiation, λ = 0.71073 Å
Hall symbol: -P 2ybc	Cell parameters from 3900 reflections
*a* = 15.3351 (3) Å	θ = 2.5–25°
*b* = 8.1327 (2) Å	µ = 0.10 mm^−^^1^
*c* = 14.5308 (3) Å	*T* = 293 K
β = 117.387 (1)°	Block, colourless
*V* = 1609.10 (6) Å^3^	0.32 × 0.28 × 0.25 mm
*Z* = 4	

### Data collection

**Table:**

Bruker Kappa APEXII CCD diffractometer	3900 reflections with *I* > 2σ(*I*)
Radiation source: fine-focus sealed tube	*R*_int_ = 0.032
graphite	θ_max_ = 33.2°, θ_min_ = 2.8°
ω and φ scans	*h* = −23→23
23127 measured reflections	*k* = −12→11
6121 independent reflections	*l* = −22→22

### Refinement

**Table:**

Refinement on *F*^2^	Primary atom site location: structure-invariant direct methods
Least-squares matrix: full	Secondary atom site location: difference Fourier map
*R*[*F*^2^ > 2σ(*F*^2^)] = 0.058	Hydrogen site location: inferred from neighbouring sites
*wR*(*F*^2^) = 0.196	H-atom parameters constrained
*S* = 1.03	*w* = 1/[σ^2^(*F*_o_^2^) + (0.1083*P*)^2^ + 0.189*P*] where *P* = (*F*_o_^2^ + 2*F*_c_^2^)/3
6121 reflections	(Δ/σ)_max_ = 0.002
216 parameters	Δρ_max_ = 0.41 e Å^−^^3^
1 restraint	Δρ_min_ = −0.25 e Å^−^^3^

### Special details

**Table:**

Geometry. All e.s.d.'s (except the e.s.d. in the dihedral angle between two l.s. planes) are estimated using the full covariance matrix. The cell e.s.d.'s are taken into account individually in the estimation of e.s.d.'s in distances, angles and torsion angles; correlations between e.s.d.'s in cell parameters are only used when they are defined by crystal symmetry. An approximate (isotropic) treatment of cell e.s.d.'s is used for estimating e.s.d.'s involving l.s. planes.
Refinement. Refinement of *F*^2^ against ALL reflections. The weighted *R*-factor *wR* and goodness of fit *S* are based on *F*^2^, conventional *R*-factors *R* are based on *F*, with *F* set to zero for negative *F*^2^. The threshold expression of *F*^2^ > σ(*F*^2^) is used only for calculating *R*-factors(gt) *etc*. and is not relevant to the choice of reflections for refinement. *R*-factors based on *F*^2^ are statistically about twice as large as those based on *F*, and *R*- factors based on ALL data will be even larger.

### Fractional atomic coordinates and isotropic or equivalent isotropic displacement parameters (Å^2^)

**Table:**

	*x*	*y*	*z*	*U*_iso_*/*U*_eq_	Occ. (<1)
C1A	0.640 (2)	0.888 (2)	0.458 (2)	0.117 (3)	0.20 (3)
H1A1	0.6596	0.8863	0.4043	0.175*	0.20 (3)
H1A2	0.6970	0.8979	0.5243	0.175*	0.20 (3)
H1A3	0.5974	0.9805	0.4479	0.175*	0.20 (3)
C1B	0.6664 (8)	0.834 (2)	0.4528 (4)	0.117 (3)	0.80 (3)
H1B1	0.6854	0.7851	0.4044	0.175*	0.80 (3)
H1B2	0.7218	0.8351	0.5206	0.175*	0.80 (3)
H1B3	0.6444	0.9445	0.4316	0.175*	0.80 (3)
C2	0.50275 (11)	0.7283 (2)	0.36557 (11)	0.0484 (4)	
C3	0.48701 (12)	0.7903 (2)	0.27035 (12)	0.0532 (4)	
H3	0.5369	0.8465	0.2643	0.064*	
C4	0.39705 (12)	0.7686 (2)	0.18441 (11)	0.0477 (4)	
H4	0.3870	0.8102	0.1207	0.057*	
C5	0.32155 (9)	0.68542 (15)	0.19185 (9)	0.0332 (3)	
C6	0.33683 (9)	0.62281 (13)	0.28794 (8)	0.0278 (2)	
C7	0.42728 (10)	0.64540 (17)	0.37363 (9)	0.0372 (3)	
H7	0.4377	0.6044	0.4376	0.045*	
C8	0.25554 (8)	0.53368 (14)	0.29997 (8)	0.0279 (2)	
H8	0.2826	0.5052	0.3735	0.034*	
C9	0.22779 (9)	0.37267 (14)	0.24099 (9)	0.0310 (2)	
C10	0.28657 (13)	0.23670 (18)	0.28828 (12)	0.0516 (4)	
H10	0.3393	0.2477	0.3541	0.062*	
C11	0.26797 (18)	0.0857 (2)	0.23924 (16)	0.0808 (7)	
H11	0.3072	−0.0042	0.2728	0.097*	
C12	0.19121 (17)	0.0676 (2)	0.14040 (16)	0.0775 (7)	
H12	0.1782	−0.0332	0.1064	0.093*	
C13	0.13489 (12)	0.20355 (17)	0.09422 (12)	0.0478 (4)	
C14	0.15003 (9)	0.35523 (14)	0.14216 (10)	0.0335 (2)	
H14	0.1091	0.4436	0.1090	0.040*	
C15	0.16393 (11)	0.7236 (2)	0.35184 (10)	0.0447 (3)	
C16	0.07682 (16)	0.8327 (3)	0.32137 (16)	0.0799 (7)	
H16A	0.0179	0.7684	0.2885	0.120*	
H16B	0.0768	0.9151	0.2740	0.120*	
H16C	0.0797	0.8848	0.3820	0.120*	
N1	0.17154 (8)	0.64005 (14)	0.27656 (8)	0.0348 (2)	
H1	0.1263	0.6493	0.2136	0.042*	
N2	0.05722 (11)	0.18961 (17)	−0.01317 (11)	0.0577 (4)	
O1	0.58945 (9)	0.7410 (2)	0.45516 (10)	0.0795 (5)	
O2	0.23072 (7)	0.66466 (13)	0.11020 (7)	0.0423 (2)	
H2	0.2299	0.7038	0.0578	0.063*	
O3	0.05139 (15)	0.06269 (19)	−0.06005 (12)	0.1014 (7)	
O4	0.00217 (11)	0.30376 (18)	−0.05160 (11)	0.0816 (5)	
O5	0.22599 (10)	0.7079 (2)	0.44251 (9)	0.0746 (5)	

### Atomic displacement parameters (Å^2^)

**Table:**

	*U*^11^	*U*^22^	*U*^33^	*U*^12^	*U*^13^	*U*^23^
C1A	0.050 (3)	0.233 (7)	0.0560 (14)	−0.069 (5)	0.0140 (19)	0.011 (3)
C1B	0.050 (3)	0.233 (7)	0.0560 (14)	−0.069 (5)	0.0140 (19)	0.011 (3)
C2	0.0342 (7)	0.0719 (10)	0.0316 (6)	−0.0132 (6)	0.0086 (6)	0.0049 (6)
C3	0.0426 (8)	0.0758 (11)	0.0417 (8)	−0.0160 (7)	0.0199 (7)	0.0096 (7)
C4	0.0465 (8)	0.0653 (9)	0.0314 (6)	−0.0103 (7)	0.0180 (6)	0.0115 (6)
C5	0.0347 (6)	0.0399 (6)	0.0226 (5)	−0.0013 (5)	0.0111 (5)	0.0041 (4)
C6	0.0304 (5)	0.0314 (5)	0.0214 (4)	0.0003 (4)	0.0118 (4)	0.0018 (4)
C7	0.0344 (6)	0.0493 (7)	0.0238 (5)	−0.0044 (5)	0.0100 (5)	0.0049 (5)
C8	0.0300 (5)	0.0332 (5)	0.0188 (4)	−0.0004 (4)	0.0098 (4)	0.0001 (3)
C9	0.0334 (6)	0.0320 (5)	0.0252 (5)	−0.0019 (4)	0.0116 (5)	0.0010 (4)
C10	0.0575 (9)	0.0393 (7)	0.0350 (7)	0.0069 (6)	0.0017 (7)	0.0016 (5)
C11	0.0947 (16)	0.0388 (8)	0.0589 (11)	0.0212 (9)	−0.0076 (11)	−0.0013 (7)
C12	0.0909 (15)	0.0358 (7)	0.0598 (11)	0.0108 (8)	−0.0046 (10)	−0.0117 (7)
C13	0.0477 (8)	0.0378 (6)	0.0380 (7)	0.0005 (6)	0.0026 (6)	−0.0079 (5)
C14	0.0328 (6)	0.0327 (5)	0.0294 (5)	0.0003 (4)	0.0095 (5)	−0.0018 (4)
C15	0.0397 (7)	0.0641 (9)	0.0296 (6)	0.0056 (6)	0.0154 (6)	−0.0126 (6)
C16	0.0686 (13)	0.1103 (17)	0.0559 (11)	0.0371 (12)	0.0245 (10)	−0.0197 (11)
N1	0.0314 (5)	0.0479 (6)	0.0216 (4)	0.0042 (4)	0.0092 (4)	−0.0058 (4)
N2	0.0559 (8)	0.0492 (7)	0.0434 (7)	0.0002 (6)	0.0016 (6)	−0.0162 (6)
O1	0.0415 (7)	0.1398 (13)	0.0397 (6)	−0.0371 (8)	0.0038 (5)	0.0145 (7)
O2	0.0397 (5)	0.0583 (6)	0.0221 (4)	−0.0068 (4)	0.0085 (4)	0.0098 (4)
O3	0.1210 (15)	0.0656 (9)	0.0623 (9)	0.0099 (9)	−0.0052 (9)	−0.0328 (7)
O4	0.0698 (9)	0.0699 (8)	0.0517 (7)	0.0233 (7)	−0.0179 (7)	−0.0195 (6)
O5	0.0692 (9)	0.1152 (11)	0.0278 (5)	0.0295 (8)	0.0124 (6)	−0.0204 (6)

### Geometric parameters (Å, °)

**Table:**

C1A—O1	1.416 (4)	C9—C14	1.3888 (17)
C1A—H1A1	0.9600	C9—C10	1.3920 (18)
C1A—H1A2	0.9600	C10—C11	1.382 (2)
C1A—H1A3	0.9600	C10—H10	0.9300
C1B—O1	1.416 (4)	C11—C12	1.384 (3)
C1B—H1B1	0.9600	C11—H11	0.9300
C1B—H1B2	0.9600	C12—C13	1.373 (2)
C1B—H1B3	0.9600	C12—H12	0.9300
C2—O1	1.3714 (18)	C13—C14	1.3827 (18)
C2—C3	1.386 (2)	C13—N2	1.4685 (19)
C2—C7	1.390 (2)	C14—H14	0.9300
C3—C4	1.382 (2)	C15—O5	1.2264 (18)
C3—H3	0.9300	C15—O5	1.2264 (18)
C4—C5	1.3872 (18)	C15—N1	1.3375 (15)
C4—H4	0.9300	C15—C16	1.491 (2)
C5—O2	1.3623 (15)	C16—H16A	0.9600
C5—C6	1.4010 (15)	C16—H16B	0.9600
C6—C7	1.3854 (17)	C16—H16C	0.9600
C6—C8	1.5199 (16)	N1—H1	0.8600
C7—H7	0.9300	N2—O4	1.2052 (19)
C8—N1	1.4561 (15)	N2—O3	1.2172 (18)
C8—C9	1.5149 (16)	O2—H2	0.8200
C8—H8	0.9800		
			
O1—C1A—H1A1	109.5	C11—C10—C9	121.21 (14)
O1—C1A—H1A2	109.5	C11—C10—H10	119.4
O1—C1A—H1A3	109.5	C9—C10—H10	119.4
O1—C1B—H1B1	109.5	C10—C11—C12	120.34 (15)
O1—C1B—H1B2	109.5	C10—C11—H11	119.8
H1B1—C1B—H1B2	109.5	C12—C11—H11	119.8
O1—C1B—H1B3	109.5	C13—C12—C11	117.69 (14)
H1B1—C1B—H1B3	109.5	C13—C12—H12	121.2
H1B2—C1B—H1B3	109.5	C11—C12—H12	121.2
O1—C2—C3	124.59 (13)	C12—C13—C14	123.38 (14)
O1—C2—C7	115.97 (12)	C12—C13—N2	118.51 (13)
C3—C2—C7	119.43 (13)	C14—C13—N2	118.06 (12)
C4—C3—C2	119.91 (13)	C13—C14—C9	118.51 (12)
C4—C3—H3	120.0	C13—C14—H14	120.7
C2—C3—H3	120.0	C9—C14—H14	120.7
C3—C4—C5	120.94 (12)	O5—C15—N1	120.63 (13)
C3—C4—H4	119.5	O5—C15—N1	120.63 (13)
C5—C4—H4	119.5	O5—C15—C16	121.81 (13)
O2—C5—C4	123.24 (11)	O5—C15—C16	121.81 (13)
O2—C5—C6	117.27 (11)	N1—C15—C16	117.56 (13)
C4—C5—C6	119.46 (12)	C15—C16—H16A	109.5
C7—C6—C5	119.13 (11)	C15—C16—H16B	109.5
C7—C6—C8	119.67 (9)	H16A—C16—H16B	109.5
C5—C6—C8	121.19 (10)	C15—C16—H16C	109.5
C6—C7—C2	121.12 (11)	H16A—C16—H16C	109.5
C6—C7—H7	119.4	H16B—C16—H16C	109.5
C2—C7—H7	119.4	C15—N1—C8	120.75 (11)
N1—C8—C9	113.16 (10)	C15—N1—H1	119.6
N1—C8—C6	111.96 (9)	C8—N1—H1	119.6
C9—C8—C6	112.27 (9)	O4—N2—O3	122.58 (15)
N1—C8—H8	106.3	O4—N2—C13	119.06 (12)
C9—C8—H8	106.3	O3—N2—C13	118.36 (14)
C6—C8—H8	106.3	C2—O1—C1B	118.2 (3)
C14—C9—C10	118.84 (11)	C2—O1—C1A	111.8 (10)
C14—C9—C8	123.88 (10)	C5—O2—H2	109.5
C10—C9—C8	117.24 (11)		
			
O1—C2—C3—C4	179.06 (19)	C9—C10—C11—C12	1.5 (4)
C7—C2—C3—C4	−0.4 (3)	C10—C11—C12—C13	−0.4 (4)
C2—C3—C4—C5	0.2 (3)	C11—C12—C13—C14	−1.3 (4)
C3—C4—C5—O2	178.11 (15)	C11—C12—C13—N2	176.0 (2)
C3—C4—C5—C6	0.1 (2)	C12—C13—C14—C9	1.9 (3)
O2—C5—C6—C7	−178.18 (11)	N2—C13—C14—C9	−175.45 (14)
O2—C5—C6—C8	0.70 (17)	C10—C9—C14—C13	−0.8 (2)
C4—C5—C6—C8	178.87 (12)	C8—C9—C14—C13	176.87 (13)
C5—C6—C7—C2	−0.3 (2)	O5—C15—N1—C8	−2.4 (2)
C8—C6—C7—C2	−179.14 (13)	O5—C15—N1—C8	−2.4 (2)
O1—C2—C7—C6	−179.05 (15)	C16—C15—N1—C8	178.72 (17)
C3—C2—C7—C6	0.5 (2)	C9—C8—N1—C15	138.63 (13)
C7—C6—C8—N1	116.41 (12)	C6—C8—N1—C15	−93.27 (14)
C5—C6—C8—N1	−62.46 (13)	C12—C13—N2—O4	173.9 (2)
C7—C6—C8—C9	−115.02 (12)	C14—C13—N2—O4	−8.6 (3)
C5—C6—C8—C9	66.11 (14)	C12—C13—N2—O3	−6.4 (3)
N1—C8—C9—C14	32.10 (15)	C14—C13—N2—O3	171.07 (19)
C6—C8—C9—C14	−95.83 (14)	C3—C2—O1—C1B	4.8 (10)
N1—C8—C9—C10	−150.24 (13)	C7—C2—O1—C1B	−175.7 (9)
C6—C8—C9—C10	81.83 (14)	C3—C2—O1—C1A	32.3 (18)
C14—C9—C10—C11	−0.8 (3)	C7—C2—O1—C1A	−148.2 (18)
C8—C9—C10—C11	−178.63 (19)		

### Hydrogen-bond geometry (Å, °)

**Table:**

*D*—H···*A*	*D*—H	H···*A*	*D*···*A*	*D*—H···*A*
O2—H2···O5^i^	0.82	1.80	2.617 (2)	179
N1—H1···O4^ii^	0.86	2.30	3.159 (2)	174
C10—H10···O1^iii^	0.93	2.47	3.320 (2)	152
C12—H12···O2^iv^	0.93	2.58	3.397 (2)	147
C14—H14···O4^ii^	0.93	2.55	3.470 (2)	169
C8—H8···O5	0.98	2.30	2.714 (2)	105
C11—H11···Cg1^iv^	0.93	2.83	3.680 (2)	153
C1B—H1B1···Cg2^v^	0.96	2.61	3.531 (2)	160

Symmetry codes: (i) *x*, −*y*+3/2, *z*−1/2; (ii) −*x*, −*y*+1, −*z*; (iii) −*x*+1, −*y*+1, −*z*+1; (iv) *x*, *y*−1, *z*;  (v) −*x*+1, *y*+1/2, −*z*+1/2.
